# A neural model of human fear pathways based on anatomical and neuroimaging data

**DOI:** 10.1186/1471-2202-12-S1-P241

**Published:** 2011-07-18

**Authors:** David Silverstein, Anders Lansner, Martin Ingvar, Arne Öhman

**Affiliations:** 1 Dept. of Computational Biology, Royal Institute of Technology, Stockholm, Sweden; 2 Dept. of Clinical Neuroscience, Karolinska Institutet, Stockholm, Sweden; 3 Stockholm Brain Institute, Stockholm, Sweden

## 

Fear responses in the human brain consist of several evolved pathways with different levels of processing [1].

A neural model has been developed for human fear, which includes a subcortical (low) road and cortical (high) road for affective processing and cortical control. The neural model is at a mid to high level of abstraction, at the level of neural populations. Each brain region of interest (ROI) in the model is represented by one or more neural populations. Neurons in these populations are non-spiking, adapting and can have both excitatory and inhibitory connections. A neural activity level (mean spiking rate) between 0 and 1 was recorded for every population within an ROI. This output was convolved with a canonical hemodynamic response function (HRF) to produce a synthetic BOLD signal for comparison with PET and fMRI experimental data. To compare model simulations with a fear/phobic PET study [2], event and timing protocols were mapped to corresponding model inputs, which included both masked and long presentations of fear relevant and neutral images for phobic and non-phobic subjects. The thalamus, visual cortex (2 levels), amygdala, orbitofrontal cortex (OFC) and prefrontal cortex (PFC) are represented in the model as ROIs. Several recurrent loops are represented, including visual cortical-thalamic and PFC-OFC loops. The amygdala has neural populations for the intercalated cells (ITC), lateral and central nuclei. Amygdala inputs entering the lateral nuclei are modulated by the ITC before producing output from the central nuclei. The phobic state was simulated by reducing the strength of the recurrent connections between the neural populations involved in a PFC-OFC loop, resulting in less inhibition of the amygdala via the ITC. Simulation results are presented that show activation of neural populations over simulated trials which correspond with ROI activity in the fear PET study.

**Figure 1:**
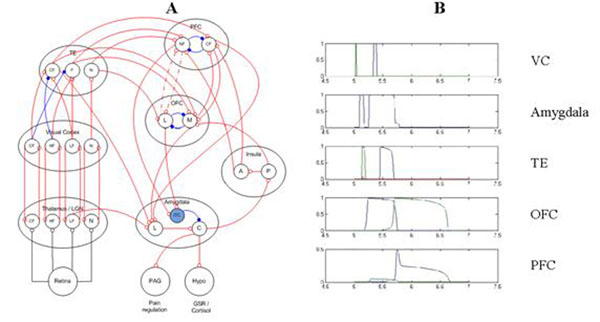
Fear network model and simulation results. **A.** Neural non-spiking population model with 7 ROIs.**B.** Neural population mean-field levels from a simulation of a masked, long (274ms) fear-relevant stimulus.
